# Supplementary material for: A Novel Exercise for Enhancing Visuospatial Ability in Older Adults with Frailty: Development, Feasibility, and Effectiveness
Source: Geriatrics (Basel). 2020 May 3;5(2):29. doi: 10.3390/geriatrics5020029 (PMC7345634; doi:10.3390/geriatrics5020029)
Supplement: Supplementary file 1 [file geriatrics-05-00029-s001.zip › supplementary file_2.pptx]

## Slide 1
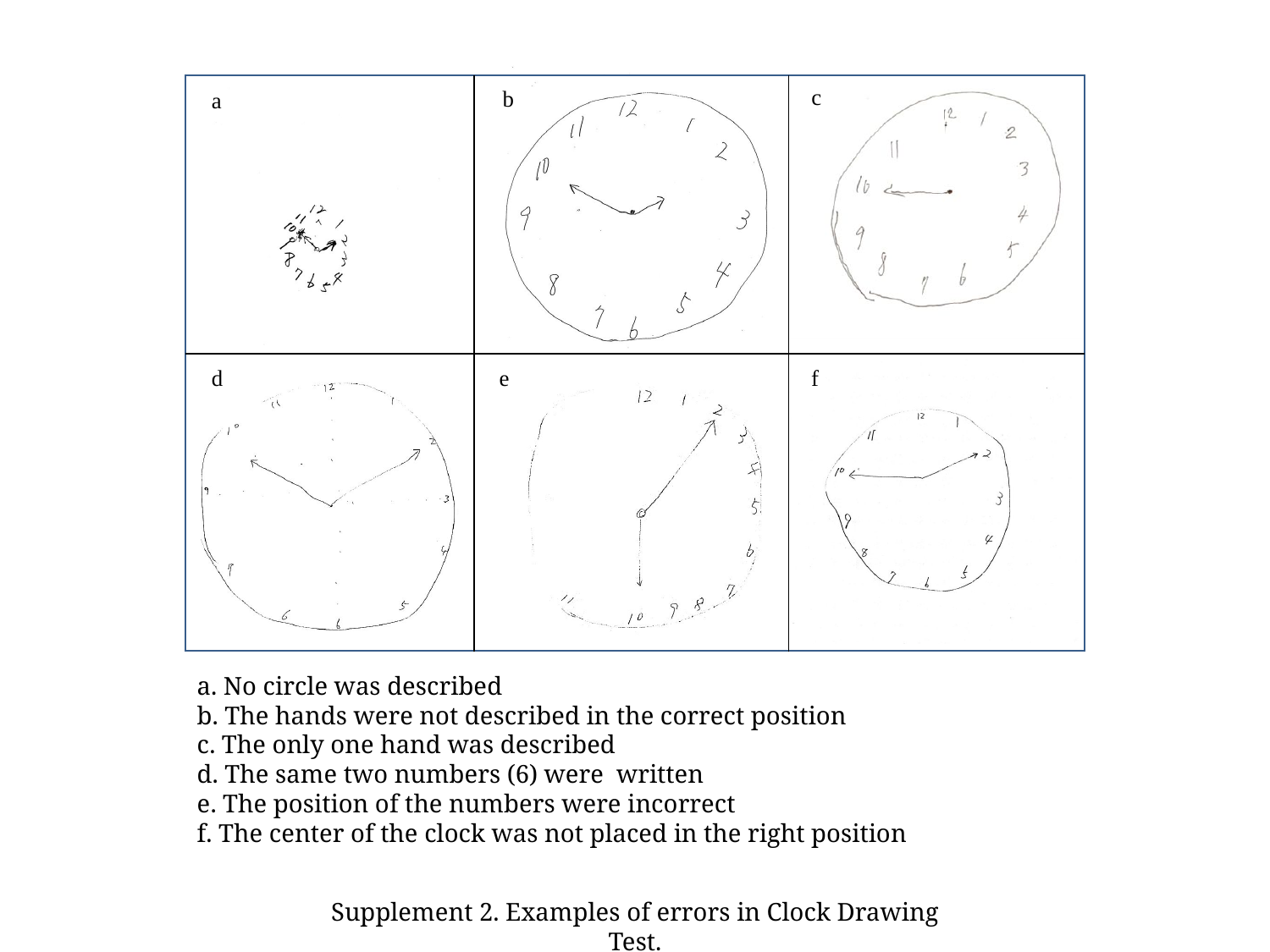

a. No circle was described
b. The hands were not described in the correct position
c. The only one hand was described
d. The same two numbers (6) were written
e. The position of the numbers were incorrect
f. The center of the clock was not placed in the right position
Supplement 2. Examples of errors in Clock Drawing Test.
