# Supplementary material for: A Novel Exercise for Enhancing Visuospatial Ability in Older Adults with Frailty: Development, Feasibility, and Effectiveness
Source: Geriatrics (Basel). 2020 May 3;5(2):29. doi: 10.3390/geriatrics5020029 (PMC7345634; doi:10.3390/geriatrics5020029)
Supplement: Supplementary file 1 [file geriatrics-05-00029-s001.zip › supplementary file_1.pptx]

## Slide 1
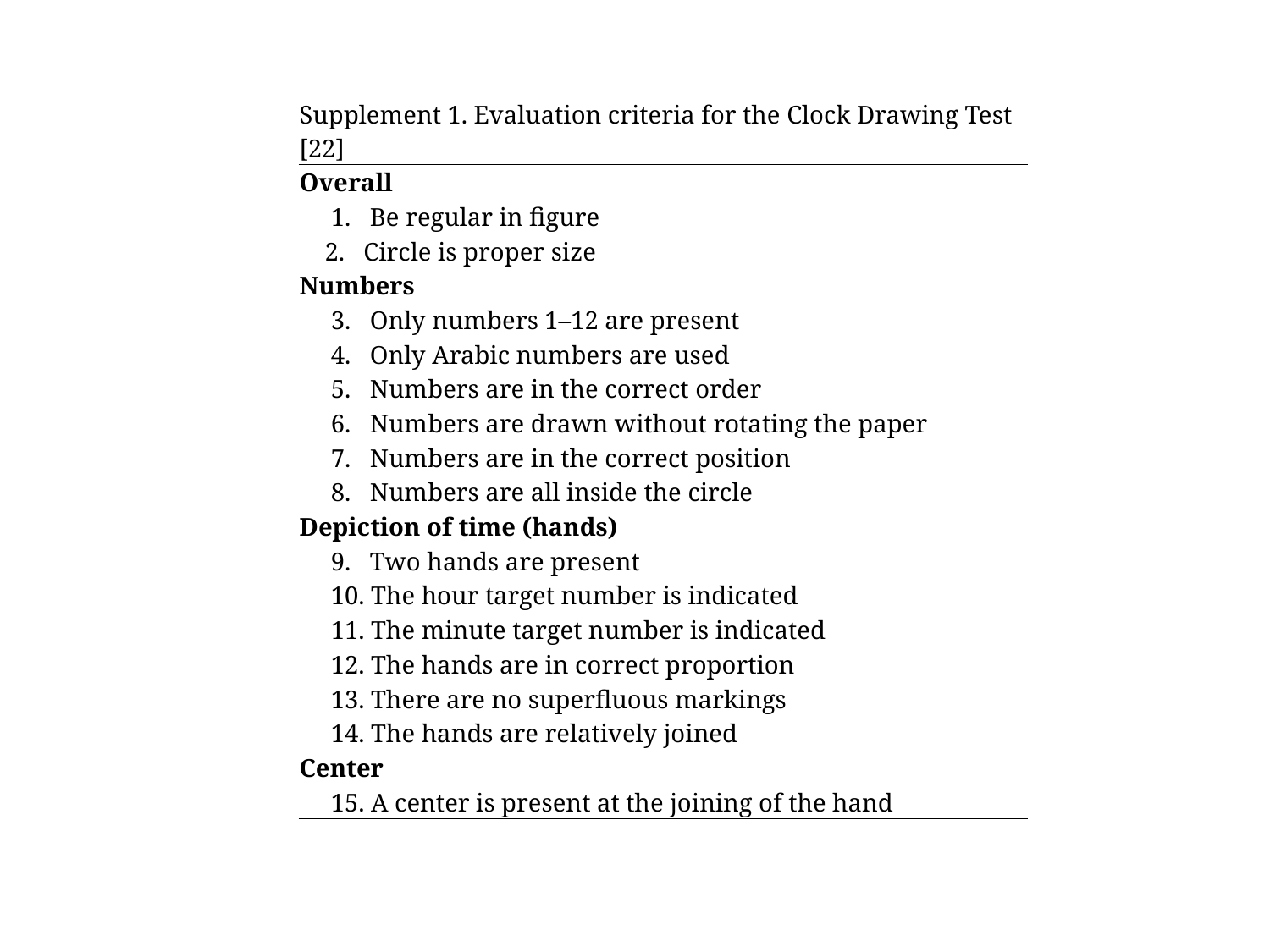

| Supplement 1. Evaluation criteria for the Clock Drawing Test [22] |
| --- |
| Overall |
| 1. Be regular in figure |
| 2. Circle is proper size |
| Numbers |
| 3. Only numbers 1–12 are present |
| 4. Only Arabic numbers are used |
| 5. Numbers are in the correct order |
| 6. Numbers are drawn without rotating the paper |
| 7. Numbers are in the correct position |
| 8. Numbers are all inside the circle |
| Depiction of time (hands) |
| 9. Two hands are present |
| 10. The hour target number is indicated |
| 11. The minute target number is indicated |
| 12. The hands are in correct proportion |
| 13. There are no superfluous markings |
| 14. The hands are relatively joined |
| Center |
| 15. A center is present at the joining of the hand |
| |
