# Supplementary material for: A Novel Exercise for Enhancing Visuospatial Ability in Older Adults with Frailty: Development, Feasibility, and Effectiveness
Source: Geriatrics (Basel). 2020 May 3;5(2):29. doi: 10.3390/geriatrics5020029 (PMC7345634; doi:10.3390/geriatrics5020029)
Supplement: Supplementary file 1 [file geriatrics-05-00029-s001.zip › supplementary file_3.pptx]

## Slide 1
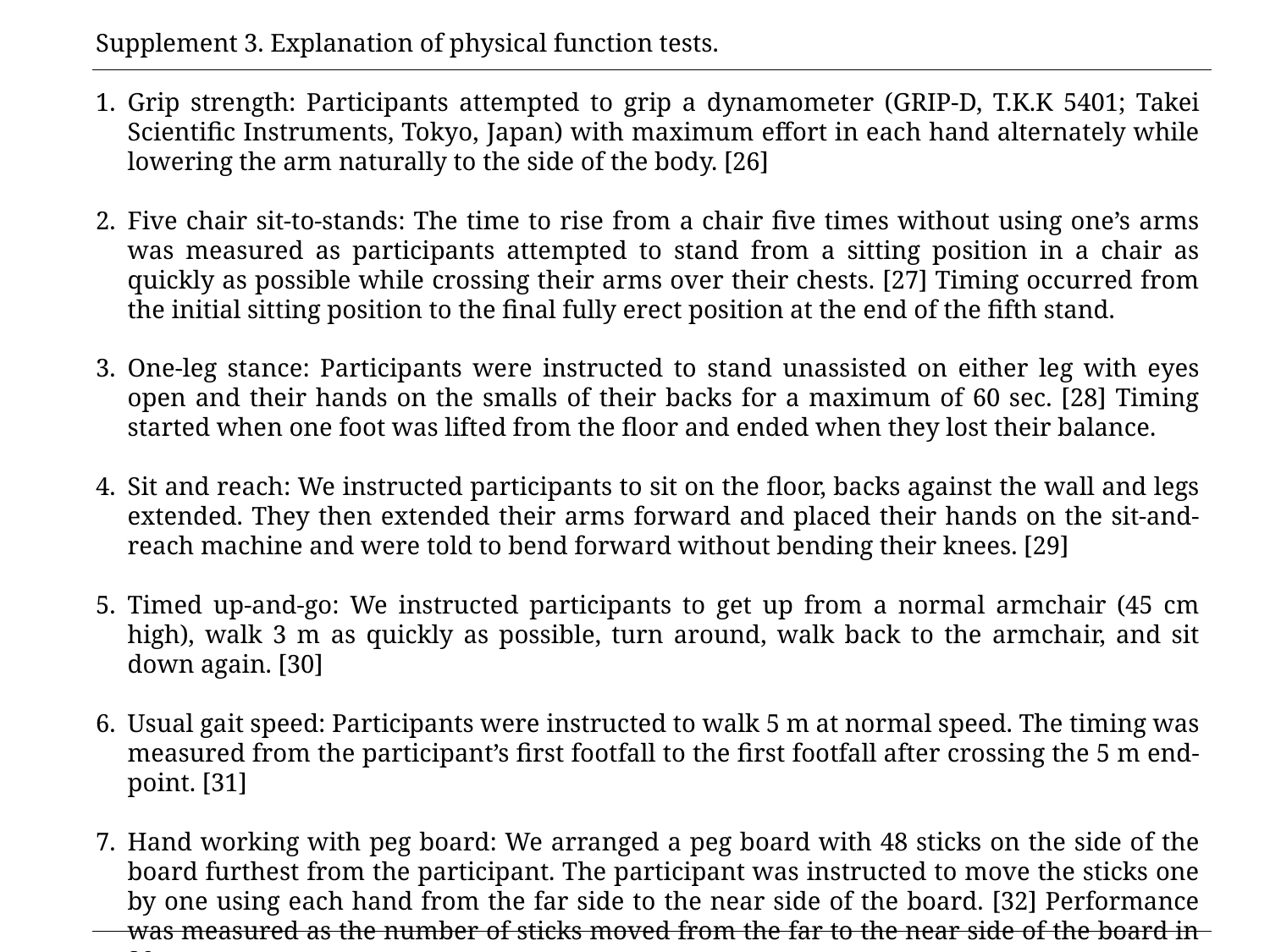

Supplement 3. Explanation of physical function tests.
Grip strength: Participants attempted to grip a dynamometer (GRIP-D, T.K.K 5401; Takei Scientific Instruments, Tokyo, Japan) with maximum effort in each hand alternately while lowering the arm naturally to the side of the body. [26]
Five chair sit-to-stands: The time to rise from a chair five times without using one’s arms was measured as participants attempted to stand from a sitting position in a chair as quickly as possible while crossing their arms over their chests. [27] Timing occurred from the initial sitting position to the final fully erect position at the end of the fifth stand.
One-leg stance: Participants were instructed to stand unassisted on either leg with eyes open and their hands on the smalls of their backs for a maximum of 60 sec. [28] Timing started when one foot was lifted from the floor and ended when they lost their balance.
Sit and reach: We instructed participants to sit on the floor, backs against the wall and legs extended. They then extended their arms forward and placed their hands on the sit-and-reach machine and were told to bend forward without bending their knees. [29]
Timed up-and-go: We instructed participants to get up from a normal armchair (45 cm high), walk 3 m as quickly as possible, turn around, walk back to the armchair, and sit down again. [30]
Usual gait speed: Participants were instructed to walk 5 m at normal speed. The timing was measured from the participant’s first footfall to the first footfall after crossing the 5 m end-point. [31]
Hand working with peg board: We arranged a peg board with 48 sticks on the side of the board furthest from the participant. The participant was instructed to move the sticks one by one using each hand from the far side to the near side of the board. [32] Performance was measured as the number of sticks moved from the far to the near side of the board in 30 sec.
